# Supplementary material for: Navigating vaccination choices: The intersecting dynamics of institutional trust, belonging and message perception among Congolese migrants in London, UK (a reflexive thematic analysis)
Source: PLOS Glob Public Health. 2024 Jul 10;4(7):e0002620. doi: 10.1371/journal.pgph.0002620 (PMC11236099; doi:10.1371/journal.pgph.0002620)
Supplement: Attachment — Submitted filename: Decision-letter-response.docx [file pgph.0002620.s002.docx]

The Migrant Health Research Group,

St George’s, University of London

Cranmer Terrace,

London,

UK

8th March 2024

Dear Professor Gottlieb,

We are delighted that the reviewers have recommended our manuscript for publication and sincerely appreciate the opportunity to revise it in line with their feedback.

We have thoroughly addressed all of their comments in the revised version, and feel it is much strengthened. Please find our detailed line-by-line response below. We have used tracked changes in the manuscript and included a clean version as part of our re-submission.

We look forward to hearing from you about next steps in the publication process.

Sincerely,

**Alison Crawshaw PhD**

**Sally Hargreaves PhD FRCPE**

on behalf of all of the co-authors.

Response to reviewers

Journal Requirements:

1. Please provide additional information regarding the considerations  made for the migrants included in this study. For instance, please discuss whether participants were able to opt out of the study and whether individuals who did not participate receive the same treatment offered to participants.

This has been clarified in the methods section, see lines 211-212. Treatment does not apply as there was no intervention reported.

2. Please update your online Competing Interests statement. If you have no competing interests to declare, please state: &ldquo;The authors have declared that no competing interests exist.&rdquo;

This has been updated.

3. In the online submission form, you indicated that &quot;Data are available on reasonable request from the study authors.&quot;.

All PLOS journals now require all data underlying the findings described in their manuscript to be freely available to other researchers, either 
a) In a public repository, 
b) Within the manuscript itself, or 
c) Uploaded as supplementary information.

This policy applies to all data except where public deposition would breach compliance with the protocol approved by your research ethics board. If your data cannot be made publicly available for ethical or legal reasons (e.g., public availability would compromise patient privacy), please explain your reasons by return email and your exemption request will be escalated to the editor for approval. Your exemption request will be handled independently and will not hold up the peer review process, but will need to be resolved should your manuscript be accepted for publication. One of the Editorial team will then be in touch if there are any issues.

This has been discussed with the academic editor via email.

4. Please provide separate figure files in .tif or .eps format only and remove any figures embedded in your manuscript file. Please also ensure that all files are under our size limit of 10MB. You may leave the figure captions or legends in the manuscript.

For more information about how to convert your figure files please see our guidelines: <https://journals.plos.org/globalpublichealth/s/figures>

We have written to the academic editor about this and they have agreed to forward our request to the relevant department. The software used to draw our figure can only save in .png or .tiff format.

5. Please review your reference list to ensure that it is complete and correct. If you have cited papers that have been retracted, please include the rationale for doing so in the manuscript text, or remove these references and replace them with relevant current references. Any changes to the reference list should be mentioned in the rebuttal letter that accompanies your revised manuscript. If you need to cite a retracted article, indicate the article&rsquo;s retracted status in the References list and also include a citation and full reference for the retraction notice.

This has been done; no changes have been made to the included references however the reference list has been edited to remove gaps and typos highlighted by reviewer 1.

Additional Editor Comments (if provided):


[Note: HTML markup is below. Please do not edit.]

Reviewers&#39; comments:

Reviewer&#39;s Responses to Questions

Comments to the Author

1. Does this manuscript meet PLOS Global Public Health&rsquo;s [publication criteria](https://journals.plos.org/globalpublichealth/s/criteria-for-publication)? Is the manuscript technically sound, and do the data support the conclusions? The manuscript must describe methodologically and ethically rigorous research with conclusions that are appropriately drawn based on the data presented.

Reviewer #1: Yes

Reviewer #2: Yes

2. Has the statistical analysis been performed appropriately and rigorously?

Reviewer #1: N/A

Reviewer #2: N/A

3. Have the authors made all data underlying the findings in their manuscript fully available (please refer to the Data Availability Statement at the start of the manuscript PDF file)?

The [PLOS Data policy](https://journals.plos.org/globalpublichealth/s/data-availability) requires authors to make all data underlying the findings described in their manuscript fully available without restriction, with rare exception. The data should be provided as part of the manuscript or its supporting information, or deposited to a public repository. For example, in addition to summary statistics, the data points behind means, medians and variance measures should be available. If there are restrictions on publicly sharing data&mdash;e.g. participant privacy or use of data from a third party&mdash;those must be specified.

Reviewer #1: Yes

Reviewer #2: No

4. Is the manuscript presented in an intelligible fashion and written in standard English?

PLOS Global Public Health does not copyedit accepted manuscripts, so the language in submitted articles must be clear, correct, and unambiguous. Any typographical or grammatical errors should be corrected at revision, so please note any specific errors here.

Reviewer #1: Yes

Reviewer #2: Yes

5. Review Comments to the Author

Please use the space provided to explain your answers to the questions above. You may also include additional comments for the author, including concerns about dual publication, research ethics, or publication ethics. (Please upload your review as an attachment if it exceeds 20,000 characters)

Reviewer #1: Thank you for the opportunity to review this manuscript, which reports on rich qualitative data from a participatory study with Congolese migrants in the UK, and generates a novel theoretical model for engagement with health protective behaviours in this group.

Thank you very much for taking the time to review our manuscript and providing your thoughtful and constructive comments to strengthen it for publication.

The authors could consider including a short section in the introduction on participatory methods, to provide context for those less familiar with their approach. In particular, it would be useful to explain why this approach is most appropriate when working with marginalised communities such as migrants.

Thank you for this suggestion. This has been added to the methods (where we felt it fit better), please see lines 167-171.

It may also be useful to provide a little more detail on work leading up to this study, in particular the CBPR project that it is nested within, and the context in terms of co-designing an intervention with the community. This will also help clarify the specific aim of this study compared with other wider goals of the CBPR project. I would suggest including this at the end of the Introduction, so that the study-specific aim leads directly into the Methods.

Thanks - this was previously omitted due to word count. We have added a few lines and felt they fit better in the first part of the methods. We have also linked to our two other papers from this study which can provide further information, particularly around the CBPR approach. See lines 170-175.

I also wonder if the paper could be strengthened by the addition of a Box/Figure/Table summarising the key recommendations that can be made as a result of this study. These are included and explained clearly in the Discussion, but drawing them out would help guide the reader to the direct policy implications of the research.

Thanks for the suggestion – reviewer 2 also made suggestions to strengthen the recommendations. As such, we have made the existing recommendations more specific and targeted to guide the reader and facilitate uptake. Please see lines 698-716 and 749-755 (we opted not to include a new table to avoid repetition).

Some minor alterations:
&bull; Lines 189-193: add initials for each researcher. Done
&bull; Table 2: Add &lsquo;No&rsquo; as well as &lsquo;Yes&rsquo; under &lsquo;Currently have children &lt;16 years of age living in household&rsquo;, for consistency with the rest of the table. Done
&bull; Line 641 to 677: very long paragraph, consider splitting in two. This has been done in line with reviewer 2’s comments as well.
&bull; References: ref 4 needs correction, lines 887 and 888 are blank (may be an error with ref 51). Done and all references corrected.

Reviewer #2: This study is an important contribution to the field, providing a clear but nuanced description of vaccination decision-making among Congolese migrants in London. The first half of the Discussion is particularly strong, and the authors have done a great job at contextualising their findings. I have outlined some suggestions below which will hopefully improve the clarity in some sections of the paper.

Thank you very much for reviewing our manuscript and for providing such helpful and constructive comments to strengthen it for publication. We are really pleased that you feel it makes a novel and important contribution to the field.

TITLE &amp; THROUGHOUT
I suggest considering whether the title and other aspects of the paper (e.g. the Intro section of the Abstract, the study aims) should be revised to &lsquo;London&rsquo; rather than the UK. The authors mention in the Discussion that while Hackney is home to a large proportion of UK&rsquo;s Congolese migrants, the study did not explore perceptions of Congolese migrants outside of Hackney/London, and therefore labelling the study with &lsquo;UK&rsquo; suggests that the scope of the study and its aims were UK-wide.

This is a very valid point and we have updated the title as suggested.

ABSTRACT
Line 40-42: &ldquo;but has been seldom explored&rdquo;, unclear whether you&rsquo;re referring to the factors or the interventions, consider re-wording e.g. &ldquo;but these factors have been seldom explored&rdquo;. Similarly line 67-69, run-on sentence that shifts away from present progressive tense mid-sentence &ndash; consider simplifying.

Thank you, we have amended these lines as suggested.

INTRODUCTION
Line 124-130: Outlining the aims of this study and then jumping to the &lsquo;History of the DRC&rsquo; section before moving on to the Methods section is a bit odd. Perhaps flow could be improved if the &lsquo;History of the DRC&rsquo; section could be included in a Box and placed somewhere earlier on in the Introduction section (and definitely before lines 124-130) so that the aims flows directly on to the methods. Thanks – this has been updated.

METHODS
Line 172: Could the SRQR checklist please be included as a supplemental appendix?
Were the transcriptions and/or findings presented to the participants for member checking and validation?

Yes – this will be uploaded. Community partners facilitated member checking and validation of transcripts and we have added this to lines 233-234.

FINDINGS
Figure 1: It would be quicker for the reader to connect the relevant factors box with the content of the triangle if the model layout was improved, e.g. position the two closer together and use &lsquo;magnifying glass&rsquo; to connect the triangle to the relevant factors box

Thank you for your suggestions. We have tried to accommodate this although it was challenging due to the number of elements and space limitations. The diagram has been modified so that the relevant factors are now linked directly to their corresponding component in the triangle. This is hopefully a bit easier to interpret.

DISCUSSION
Lines 641-677: This paragraph is extremely long and the message of the paragraph gets lost. Consider splitting it in half at around Line 661-662 so that the first part focuses on interpretation of findings relating to institutional trust and the second half focuses on interventions/strategies/future research. Line 674 &lsquo;Overall, the findings&hellip;&rsquo; also seems to fit better in the next paragraph which focuses on placing the responsibility on policy makers in improving future PH response. Thank you for the helpful suggestions. These changes have been made.

Line 679-686: The &lsquo;agents of change&rsquo; in this paragraph could be clarified e.g. rather than continuing to use terms like policymakers and practitioners, you could be specific e.g. is it the primary care networks&rsquo; and integrated care boards&rsquo; responsibility to evaluate/understand the needs of the community, or is it the responsibility of GP practices and other vaccine service providers? Similarly, who in government should &lsquo;shoulder the responsibility&rsquo; of trust-building? From my experience, statements like this are often overlooked when they are presented to government agencies because it&rsquo;s such a high-level and non-specific ask. For example in the UK context, should it start with key vaccine-related departments within peak bodies like UKHSA, DHSC, OHID and NHS England deploying resources to regional teams to then commission programmes of work from local (and more trusted) organisations? Of course the discussion cannot be very specific in this regard, but asking yourselves these questions like this may help you to think through your recommendations in more detail and target the message in this paragraph.

Thank you for the very helpful comments and reflection based on your own experience. We have taken them on board and made these messages more specific, see lines 692-704.

CONCLUSION
Following on from my comment above, consider how the final sentences can be a bit more targeted rather than saying &lsquo;governments&rsquo; must do X, Y, Z.

Thank you, this has been made specific; see lines 734-736.

6. PLOS authors have the option to publish the peer review history of their article ([what does this mean?](https://journals.plos.org/globalpublichealth/s/editorial-and-peer-review-process#loc-peer-review-history)). If published, this will include your full peer review and any attached files.


Do you want your identity to be public for this peer review? If you choose &ldquo;no&rdquo;, your identity will remain anonymous but your review may still be made public.


For information about this choice, including consent withdrawal, please see our [Privacy Policy](https://www.plos.org/privacy-policy).

Reviewer #1: No

Reviewer #2: No

[NOTE: If reviewer comments were submitted as an attachment file, they will be attached to this email and accessible via the submission site. Please log into your account, locate the manuscript record, and check for the action link &quot;View Attachments&quot;. If this link does not appear, there are no attachment files.]

While revising your submission, please upload your figure files to the Preflight Analysis and Conversion Engine (PACE) digital diagnostic tool, https://pacev2.apexcovantage.com/. PACE helps ensure that figures meet PLOS requirements. To use PACE, you must first register as a user. Registration is free. Then, login and navigate to the UPLOAD tab, where you will find detailed instructions on how to use the tool. If you encounter any issues or have any questions when using PACE, please email PLOS at figures@plos.org. Please note that Supporting Information files do not need this step.

<span style="font-style: italic; font-size: 90%;">In compliance with data protection regulations, you may request that we remove your personal registration details at any time. [(Remove my information/details)](https://www.editorialmanager.com/pgph/login.asp?a=r). Please contact the publication office if you have any questions.

Top of Form

Bottom of Form
